# The Relationship Between Non-Transferrin-Bound Iron (NTBI), Labile Plasma Iron (LPI), and Iron Toxicity

**DOI:** 10.3390/ijms26136433

**Published:** 2025-07-03

**Authors:** Lorena Duca, Elena Di Pierro, Natalia Scaramellini, Francesca Granata, Giovanna Graziadei

**Affiliations:** 1 SC Medicina ad Indirizzo Metabolico, Fondazione IRCCS Ca’ Granda Ospedale Maggiore Policlinico, 20122 Milan, Italy; natalia.scaramellini@unimi.it (N.S.); francesca.granata@policlinico.mi.it (F.G.); giovanna.graziadei@policlinico.mi.it (G.G.); 2 Dipartimento di Scienze Cliniche e di Comunità, Dipartimento di Eccellenza 2023–2027, Università degli Studi di Milano, 20122 Milan, Italy

**Keywords:** iron metabolism, NTBI, LPI, oxidative stress, chelation

## Abstract

Plasma non-transferrin-bound iron (NTBI) comprises multiple subspecies, classified by their composition, chemical reactivity, and susceptibility to chelation. The redox-active and chelatable fraction of NTBI is referred to as labile plasma iron (LPI). The pathophysiological significance of NTBI and LPI lies in their ability to enter cells via alternative transport pathways that are not regulated by the transferrin receptor system or by cellular iron levels. Several mechanisms have been proposed for their cellular entry, including the hijacking of divalent metal transporters and passive diffusion. This unregulated uptake can lead to iron accumulation in vulnerable tissues such as the liver and the heart. NTBI and LPI bypassing normal cellular control mechanisms can rapidly exceed the cell’s capacity to safely store excess iron, leading to toxicity. Both NTBI and LPI contribute to oxidative stress by participating in free-radical-generating reactions. However, LPI concentration in the bloodstream may be differentially affected by the mode and extent of iron overload, the presence of residual serum iron-binding activity, and the antioxidant capacity of individual sera. In summary, both NTBI and LPI contribute to iron-mediated toxicity but differ in terms of reactivity, availability, and pathogenic potential depending on the pathophysiological conditions that influence the degree of toxicity.

## 1. Introduction

Iron is an important and necessary element in numerous physiological processes, allowing the normal functioning of proteins and enzymes. Iron is essential for life due to its redox activity, but it also has the potential to become cytotoxic by catalyzing the production of reactive oxygen species (ROS) [1]. Given the multiple activities and functions regulated by iron, numerous systems are actively aimed at regulating its homeostasis, thus guaranteeing constantlevels both in the plasma and in various organs. In the plasma, iron binds to transferrin as Fe^2+^, resulting in transferrin-bound iron (TBI). When plasma iron exceeds transferrin’s binding capacity, NTBI appears. Unlike TBI, whose absorption is regulated by transferrin receptors and other iron-regulatory proteins, NTBI can enter cells freely, bypassing these regulatory mechanisms [2].

Extracellular NTBI is thought to consist of the ferric ion (Fe^3+^). Due to the poor solubility of Fe^3+^ at a physiological serum pH, NTBI must exist as iron complexed with blood serum components that facilitate its solubility and binding. Low-molecular-weight (LMW) carboxylic acids, with oxygen donor atoms, are the ligands most likely responsible for NTBI binding [3]. These interactions generate several circulating isoforms of NTBI, primarily Fe^3+^ complexes with albumin, citrate, and potentially acetate [4]. Among these, citrate exhibits the greatest affinity for Fe^3+^. Under physiological conditions, two main forms of Fe^3+^–citrate complexes dominate: monomeric and oligomeric species [5]. Ferric citrate is widely considered a major form of NTBI in plasma during physiological conditions of iron overload, preferentially binding divalent cations like calcium and magnesium, which are far more abundant than iron by more than 100-fold [6]. The citrate concentration in plasma tends to remain stable at around 100 μM. Thus, the citrate/iron ratio, and by extension the chemical nature of the iron–citrate complexes, is determined by changes in NTBI levels.

The chemical nature of NTBI is heterogeneous and includes multiple subspecies that can be classified based on composition, chemical reactivity, or chelation susceptibility [7]. A critical redox-active and chelatable fraction of NTBI is referred to as LPI [8]. LPI comprises iron complexed with citrate, albumin, and other unidentified anionic ligands [9]. The pathophysiological importance of LPI lies in its unregulated uptake by tissues, unlike TBI, which enters cells via tightly regulated transferrin receptor pathways [10]. In addition, LPI serves as a readily available iron source for bacteria compared to TBI. LPI can enter cells via divalent cation transporters or the endocytosis of associated macromolecules.

**Figure 1:**
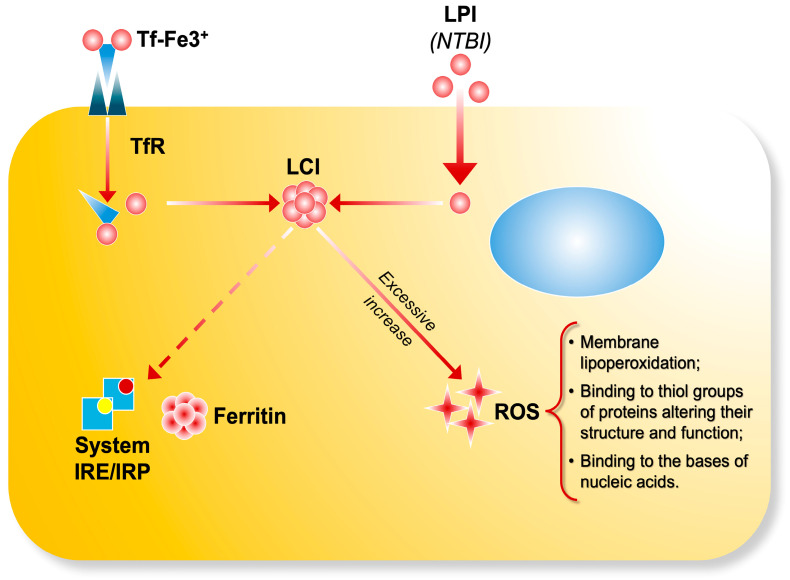
The transferrin bound iron (Tf-Fe) complex enters cells by binding to transferrin receptors and it is stored as ferritin through IRE/IRP regulation. LPI (NTBI) enters cells via unregulated pathways, contributing to the formation of labile cell iron (LCI) pools. Excessive iron uptake accumulates in the cells and catalyzes the production ofreactive oxygen species, leading to oxidative damage.

LPI consists of transient Fe^2+^ and Fe^3+^ forms, whose relative abundance is influenced by the cellular redox environment, potentially involving specific iron reductases [11]. The most accessible form of LPI within cells is assumed to be localized in the cytosol as labile cell iron (LCI), which serves as a central hub of intracellular iron trafficking. This movement is regulated by iron-sensing mechanisms involving iron-sensing proteins (IRPs) [12]. Intracellular labile iron pools consist of various forms of iron with distinct functions: in mitochondria, they are precursors for iron sulfur cluster and porphyrin synthesis; in endosomes, they originate from transferrin-bound iron internalized through receptor-mediated endocytosis; and in lysosomes, they are associated with degradation products of iron-containing proteins [13]. The composition of LCI varies depending on metal concentration, the presence of chelating molecules, pH, and the cellular redox potential [2]. Most labile iron exists in the ferrous (Fe^2+^) state, which can catalyze the formation of ROS, such as hydroxyl radicals (OH^•^), via Fenton chemistry from hydrogen peroxide [14] (Figure 1).

## 2. Iron Overload, Reactive Oxygen Species, and Organ Damage

Iron homeostasis is tightly regulated to prevent both iron deficiency and excess. However, the body lacks an active mechanism to eliminate excess iron. Iron overload may occur when iron absorption regulation is disrupted (due to genetic mutations or ineffective erythropoiesis) or bypassed (e.g., red blood cell transfusions or intravenous iron therapy). From a general point of view, iron overload can be classified as primary, when based on a primary defect in the regulation of iron metabolism and its balance, or secondary, when due to hereditary or acquired diseases. Primary hemochromatosis has a hereditary basis and is characterized by mutations in the genes involved in iron metabolism, which means that iron is absorbed in excessive quantities from the intestine into the blood. Meanwhile, secondary iron overload can be the consequence of hereditary anemias (for example, thalassemia or sickle cell anemia) or acquired anemias (for example, myelodysplasia or leukemia) or can be associated with other conditions such as chronic liver disease, Friedreich’s ataxia, or excessive administration of iron orally or parenterally (transfusions, intravenous or intramuscular iron preparations, etc.) [8].

In these conditions, iron overload (IO) is caused by iron accumulation due to repeated blood transfusions or by increased absorption of nutritional iron from the gastrointestinal tract [15]. IO also occurs due to the premature destruction of erythroid cells, which may occur (a) in the bone marrow, where developing erythroid precursors undergo increased apoptosis (ineffective erythropoiesis); (b) in the reticuloendothelial system, mainly in the spleen and liver, where senescent red blood cells undergo phagocytosis by resident macrophages (extravascular hemolysis); and (c) in the peripheral blood, if pathological red blood cells undergo lysis in the blood vessels (intravascular hemolysis) [15].

All clinical conditions associated with secondary iron overload are linked to elevated NTBI levels [11]. The presence of NTBI is hypothesized to elevate the risk of developing comorbidities [16]. Indeed, the primary toxic potential of NTBI arises not from catalyzing free radical formation in plasma, but rather from the misdistribution of iron in tissues that are not physiologically equipped to handle large iron fluxes and that lack sufficient antioxidant capacity to counteract the redox activity of labile iron within intracellular compartments.

Oxidative stress is defined as an imbalance between an excess of oxidants and the insufficient capacity of the antioxidant system to neutralize free radicals. ROS are essential for cell signaling, being involved in proliferation, differentiation, apoptosis, and immune defense in various cell types [17]. The generation of ROS attracts numerous inflammatory cells and promotes the production of inflammatory cytokines, growth factors, and transcription factors. During oxidative stress, ROS levels increase within cells.

**Figure 2:**
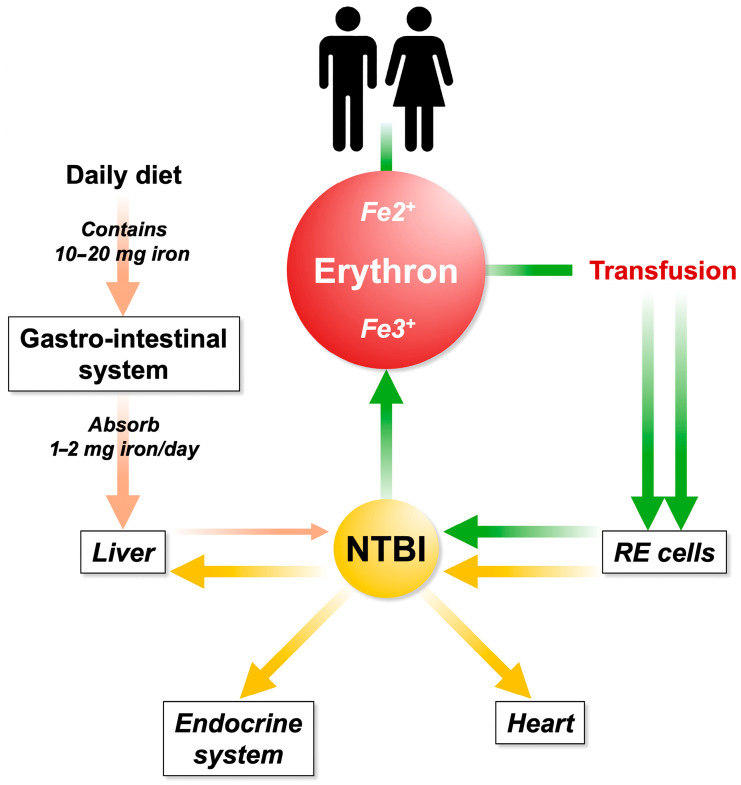
Iron is filtered through intestinal absorption and it is sent to the liver via the portal system. Excessive iron uptake is no longer bound by circulating transferrin, resulting in the formation of NTBI. Repeated and inappropriate blood transfusions contribute to iron overload and NTBI increases. The first station in the accumulation occurs in the reticuloendothelial (RE) system (spleen). Subsequently, the RE iron is recycled to the parenchymatous organs.

There is no effective mechanism to eliminate excess iron, leading to its accumulation in various organs, including vital ones such as the heart, endocrine system, and liver, causing organ damage. IO-related damage is mediated by free radicals, such as reactive oxygen species [18]. The liver is considered the primary target of iron toxicity due to its central role in iron metabolism [19]. However, while excess iron can damage all tissues, only certain organs appear to be the main targets of iron-mediated injury. It remains unclear whether this organ-specific damage is more closely related to the absolute amount of free iron capable of generating reactive oxygen species or to the duration of tissue exposure to ROS (Figure 2).

## 3. NTBI and LPI: Key Factors in the Pathogenesis of Iron-Related Diseases

### 3.1. Hemolytic Anemia

Thalassemia is a hereditary hemolytic anemia characterized by partial or complete failure to synthesize the α- or β-globin chains of adult hemoglobin (HbA), a tetramer composed of α_2_β_2_ chains. This defect results from numerous mutations in the corresponding globin genes. The imbalance leads to an excess of unpaired globin chains, which are unstable and precipitate within cells, causing the premature destruction of erythroid precursors in the bone marrow and extravascular hemolysis of mature RBCs. Degradation products of hemoglobin, including heme and iron, further exacerbate oxidative stress through the generation of oxygen free radicals [20].

β-thalassemia, characterized by reduced or absent β-globin synthesis, and myelodysplastic syndrome (MDS), where clonal stem cell mutations result in ineffective and dysplastic hematopoiesis, are both disorders marked by ineffective erythropoiesis. In these conditions, a heightened number of erythroid precursors attempt to compensate for the underlying defect but fail to mature adequately into functional red blood cells [21].

Recent in vitro and in vivo studies have demonstrated that excess iron negatively affects erythropoiesis and may worsen bone marrow failure, particularly in myelodysplastic syndrome. Regular transfusion therapy leads to increased NTBI levels in the bloodstream. Moreover, IO occurs in β-thalassemia, with blood transfusion being the major cause of transfusion-dependent thalassemia (TDT), whereas increased gastrointestinal absorption is more significant in non-transfusion-dependent thalassaemia (NTDT) [22]

The accumulation of iron from intestinal absorption in NTDT patients is slower than that observed in transfusion-associated siderosis, reaching approximately 3–4 mg/day or up to 1000 mg/year [23]. Clinical studies including NTDT patients receiving a placebo observed a mean annual increase in liver iron concentration of 0.38 ± 0.49 mg Fe/g dry weight [24]. Nonetheless, iron overload in NTDT patients is a cumulative process, as demonstrated by longitudinal studies [25] and cross-sectional studies documenting positive correlations between iron overload indices and advanced age [26]. Thus, a considerable proportion of NTDT patients eventually accumulate enough iron to reach liver iron concentration thresholds of clinical significance [24] and may begin to experience iron-related morbidity beyond 10 years of age [23]. Iron accumulation can damage multiple tissues, causing heart failure, cirrhosis, liver cancer, growth retardation, and various endocrine abnormalities [20].

### 3.2. Iron Metabolism in Hemodialysis Patients Treated by Intravenous Iron Supplementation: Labile Plasma Iron in Parenteral Iron Formulations and Its Potential Generation of Non-Transferrin-Bound Iron

The kidneys, vital organs responsible for excretion, can become impaired if damaged by iron overload. The kidneys play a vital role in maintaining homeostasis by regulating plasma components, including electrolytes and water, and by eliminating potentially harmful metabolic wastes and foreign substances. These wastes must be eliminated in solution, as they cannot be eliminated in solid form. Consequently, the kidneys must produce at least 500 mL of urine containing waste products per day [27]. Iron resists dissociation and can cause oxidative cellular damage [28].

In hemodialysis (HD) patients, iron supplementation is common to maintain adequate iron stores for erythropoiesis and to sustain proper hemoglobin levels. NTBI in HD patients may originate from both intravenous iron therapy and the hemodialysis procedure itself, possibly due to microhemolysis. Although NTBI was found to be catalytically inactive in this context, its exact nature remains unclear in the dialysis population. Evidence suggests that it is neither heme-bound iron nor ferritin-bound iron [29].

Different species of NTBI and other factors can influence the distribution of tissue hemosiderosis [30]. NTBI levels vary depending on the underlying disease and dependency on treatment. The appearance of NTBI is typically transient after oral or intravenous iron administration. In studies using oral iron doses equivalent to 100 mg of ferrous sulfate, NTBI concentrations ranged from 1 to 6 µM. Garbowski et al. [31] evaluated NTBI levels post oral iron administration and reported peak NTBI values between 0.13 and 1.25 µM (Fe-sucrose > Fe-carboxymaltose> Fe-isomaltoside-1000). They also evaluated labile plasma iron, reflecting redox-active NTBI subspecies, and found lower LPI levels than NTBI: 0.83, 0.15 and 0.18 μM for Fe-sucrose, Fe-isomaltoside-1000 and Fe-carboxymaltose, respectively [31]). NTBI’s presence after iron administration has been linked to increases in non-specific oxidative stress biomarkers [28].

Large datasets from dialysis patients have revealed a modest association between high iron doses and increased infection risk. However, this was not observed in a prospective study on chronic kidney disease patients [32]. Prakash et al. [33] found significantly higher NTBI and ferritin levels in hemodialysis patients not receiving intravenous iron therapy compared to chronic renal failure patients and healthy controls. Dresow et al. [34] first reported the occurrence of NTBI following oral iron supplementation in patients with iron deficiency anemia. Interestingly, NTBI levels were comparable to those found in iron overload disorders, even when transferrin saturation remained below 100% [35].

Esposito et al. [36] reported LPI detection in a large cohort of dialysis patients, finding only a weak correlation between LPI presence and the amount of iron received over the preceding six months. This suggests individual variability in susceptibility to LPI, possibly due to differences in iron regulation or in the properties of specific intravenous iron formulations. LPI appeared to correlate significantly with transferrin saturation, supporting the theory that it arises from iron overload [36]. Bnaya et al. [37] noted that LPI was frequently detected in patients with high plasma ferritin levels or those receiving large monthly intravenous iron doses.

Malyszko et al. [35] suggested that the increased occurrence of LPI in severe renal disease may reflect impaired iron metabolism. Whether LPI indicates “hidden” iron overload remains uncertain. They also noted that serum ferritin has limited diagnostic value, showing no correlation with LPI [35]. A univariate analysis showed correlations between NTBI and inflammatory markers, including ferritin, reinforcing the notion that iron contributes to infection and inflammation pathogenesis.

Experimental infection models have shown that iron significantly enhances bacterial virulence. The mechanisms by which potential pathogens acquire the iron required for in vivo growth and virulence expression are extremely complex. For example, Gram-negative bacteria such as Escherichia coli produce siderophores like enterobactin and aerobactin, while Pseudomonas species produce pyochelin and pyoverdine [38].

Intravenous (i.v.) iron use has become more widespread in recent years thanks to the availability of safer drugs. The latest generation of preparations, administered in high doses, are better tolerated and can correct iron deficiency even after a single administration.

Intravenous iron is currently used not only in chronic renal failure and other chronic inflammatory diseases but also in heart failure [39]. Clinical studies have identified iron, with and without anemia, in patients with chronic heart failure and reduced ejection fraction [40]. Iron deficiency in this context is diagnosed when the concentration of ferritin is <100 mg/L or the transferrin saturation is <20%, regardless of ferritin levels (though the ferritin level should still be <300 mg/L) [41]. Contributing factors include chronic inflammation, loss of appetite, reduced iron intake, antithrombotic-induced blood loss, and impaired absorption due to pharmacological effects or edema of the intestinal mucosa.

**Figure 3:**
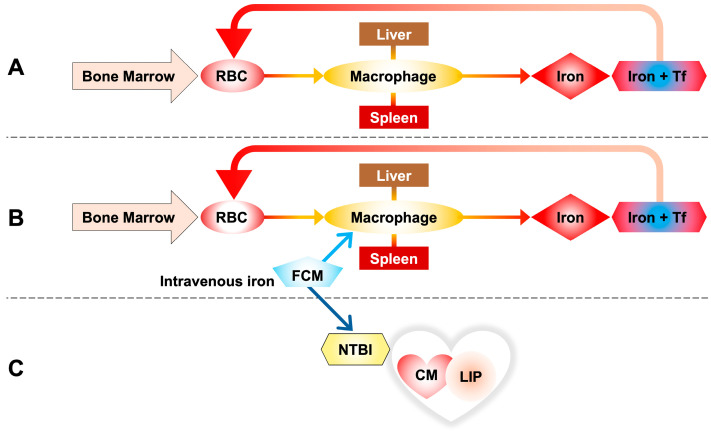
(**A**) Iron homeostasis through the phagocytosis of red blood cells (RBCs), taken up by reticuloendothelial macrophages of the spleen and liver. Iron released by macrophages is transported into the blood by transferrin to be reused for the synthesis of new hemoglobin. (**B**) Ferric carboxymaltose (FCM) is taken up by reticuloendothelial macrophages of the spleen and liver as in the standard pathway of intravenous iron metabolism. (**C**) A portion of the infused iron escapes this rule in favor of the non-standard pathway, releasing some of the iron directly into the circulation and resulting in the formation of NTBI, with direct uptake by cells, in this case cardiomyocytes.

Initial studies found that high-dose ferric carboxymaltose administration improved subjective well-being and reduced hospitalizations in heart failure patients [42]. The iron–carbohydrate complex is degraded within the macrophage lysosome, releasing iron that is exported into the circulation and captured by transferrin (Figure 3) [41].

However, repeated i.v. iron administration without accounting for long-term accumulation risks may be problematic, particularly since the body lacks a dedicated system for iron excretion.

Vera-Aviles et al. [41], using both patient data and murine/cellular models, advised caution with repeated high-dose i.v. iron due to the heart’s susceptibility to NTBI uptake and LIP accumulation. Their murine studies showed a significant increase in myocardial LIP and total iron within one hour of ferric carboxymaltose (FCM) administration. Spleen and liver iron accumulation occurred later, mirroring clinical observations.

These results bring us back to a reversal of the concept according to which parenteral iron is absorbed exclusively through the standard macrophage pathway, but it is observed that a portion of the infused iron escapes this rule in favor of the non-standard pathway that considers the presence of NTBI in circulation with direct absorption by cells, in this case cardiomyocytes (Figure 3).

## 4. NTBI and LPI in Patients with Iron Overload

### 4.1. Effects of Iron Chelation

Organ complications in patients with transfusional iron overload may be induced not only by direct parenchymal iron toxicity but also by chronic exposure to NTBI [43]. Recently, a prolonged elevation of LPI was also described to compromise organ function and negatively impact survival [44].

The concept that chronic tissue iron overload can be prevented by controlling plasma NTBI levels has led to the development of chelation regimens aimed at minimizing tissue exposure to plasma NTBI components, thereby keeping the reactive Fe^2+^ species level close to zero. Iron toxicity is related to both the amount of toxic Fe^2+^ the cell is exposed to and the duration of that exposure. This remains a critical concept in the clinical management of systemic iron overload due to tissue iron accumulation; consequently, much of the morbidity and mortality associated with systemic iron overload can be attributed to massive NTBI exposure [2]. It is therefore necessary to maintain safe iron levels in the body by balancing iron intake from blood transfusions with iron excretion through chelation [45].

The introduction of effective iron chelation therapy has been associated with improved survival, enhanced quality of life, and the remission of liver and cardiac functional complications in TDT patients. Similarly, iron chelation therapy is an indispensable option for NTDT patients with iron overload [46,47]. Since iron overload is historically a remitting condition in NTDT, only a few, mostly small, studies have evaluated the efficacy and safety of iron chelation therapy in NTDT patients.

Iron chelation therapy aims to maintain safe iron reserves in the body and to remove iron already deposited in tissues, with a faster reversal of heart failure seen in TDT than in NTDT. Three iron chelators are approved for thalassemia treatment: deferoxamine (DFO), deferiprone (DFP), and deferasirox (DFX). The evaluation of iron overload should be performed after the transfusion of 10 units of packed red blood cells in TDT patients and by the age of 10 in NTDT patients, the age at which iron-related morbidity becomes a concern [48]. The clinician should adopt an individualized approach when choosing chelation therapy, considering the overall iron overload profile, predominant organ iron deposition, transfusion requirements, likelihood of adherence, and comorbidity profile [46].

Successful chelation therapy requires the careful adjustment of frequency, duration, and dosage. Dosage reduction becomes necessary as body iron levels improve, since elevated NTBI and LPI levels can rise rapidly once the chelator is cleared from circulation [49]. Although NTBI measurements have limited value as direct indicators of iron toxicity risk, the duration for which NTBI is neutralized by chelation is vital. Existing recommendations provide a good starting point; however, the chelation regimen should ultimately be tailored based on serial assessments of liver iron concentration (LIC), magnetic resonance imaging (MRI), and serum ferritin reduction [50].

While NTBI correlates loosely with iron overload, it is also influenced by other factors such as ineffective erythropoiesis, the phase of the transfusion cycle, and the frequency of blood transfusions, complicating interpretation [46]. Furthermore, the presence of NTBI does not always correspond directly to transferrin saturation levels and may be affected by conditions unrelated to iron overload, such as infection or inflammation [51]. LPI, being susceptible to chelation, serves as a useful diagnostic indicator of iron overload and cellular toxicity when monitoring chelation efficacy [46]. Thus, the chronic control of circulating LPI levels remains a key goal in chelation therapy to prevent oxidative damage and reduce the risk of organ dysfunction [52].

Increased NTBI and LPI are also pathological concerns in myelodysplastic syndrome (MDS) and require iron chelation with the aim of achieving undetectable LPI levels [53]. Decisions about when to initiate iron chelation therapy in MDS depend on the individual patient, with the risk being damage caused by iron overload. This risk depends not only on the extent but also on the duration of iron overload. Exposure to NTBI and LPI in elderly patients with cancer, including MDS, causes greater damage than in younger patients with thalassemia. It remains unclear whether iron-mediated damage begins earlier in MDS than in thalassemia patients. Not all tissues are equally susceptible; for example, heart failure tends to develop before liver cirrhosis and is a major cause of death related to transfusion-induced iron overload [54]. The prevention of heart failure is therefore a crucial therapeutic objective, not only in children with thalassemia major but also potentially in elderly patients with MDS [55].

Importantly, several studies have demonstrated that iron chelation improves survival in transfusion-dependent MDS [56]. These studies have shown that deferasirox can reduce serum ferritin and liver iron concentration while sustaining reductions in LPI in MDS patients [57]. In the United States, median ferritin reductions were found to be greater in patients with hematologic improvements compared to those without, although no statistical differences in LPI levels were detected [58].

Cardiac iron overload, myocardial hypertrophy, and other structural effects of chronic anemia, alone or in combination, contribute to cardiac pathology, as seen for TDT. Iron-induced heart disease is responsible for 71% of mortalities in thalassemia [59,60]. This complication is preventable with intensive chelation therapy, such as the continuous infusion of DFO. The reversal of cardiac arrhythmia observed after DFO treatment has led to the concept of depleting toxic labile plasma iron becoming a primary target for chelation [61]. LPI appears when the iron-binding capacity of transferrin is exceeded due to the excessive production of catabolic iron, and it tends to recur during periods of low plasma DFO concentrations [44]. Consequently, the continuous chelation of circulating LPI may be indispensable to prevent labile iron from entering cells through pathways that bypass the tightly regulated transferrin-mediated iron uptake [62].

When DFO binds iron, forming ferrioxamine (FO), the chelated iron is primarily derived from hepatic and erythrocyte catabolism by macrophages in the spleen, liver, and bone marrow. DFO is highly efficient at chelating hepatic iron due to rapid uptake by hepatocytes [63]. FO is excreted via the urine or feces, with urinary iron mainly being derived from erythrocyte catabolism and fecal iron primarily being derived from hepatic sources. Before transfusion, the proportion of endogenous (non-transfused) red blood cells is higher, leading to greater peripheral hemolysis and ineffective erythropoiesis. This increases the pool of iron available for chelation from erythrocyte catabolism, which is reflected in higher urinary FO excretion, while fecal excretion remains relatively unchanged or slightly reduced.

Borgna-Pignatti et al. [64] reported that optimal cardioprotection is achieved through combined therapy with nocturnal subcutaneous DFO and daily oral DFP. Moreover, daily DFP monotherapy appears to be more cardioprotective than nocturnal DFO alone [65]. DFP’s ability to permeate cardiac tissues likely contributes to its cardioprotective effects. However, its use is limited by hematologic toxicity, observed in approximately 1–5% of patients [66]. Additionally, hepatic iron removal enhances myocardial iron clearance. Chelation regimens that maintain chelator presence in the plasma for 24 h offer continuous protection against NTBI and LPI uptake, which can otherwise rebound quickly after treatment cessation [44]. Nonetheless, not all forms of NTBI are equally inhibited from entering cardiomyocytes [67]. The rate of entry of a chelator and the rate at which it releases iron complexes are influenced by molecular size, charge, and lipid solubility, all of which are factors that affect the chelator’s ability to access and remove intracellular iron [23]. DFP, due to its neutral shift and low molecular weight, has a faster kinetic passage to intracellular iron stores than DFO [30]. Although the combination of chelators is theoretically beneficial through a “shuttle” mechanism, which facilitates iron removal, the clinical efficacy of this mechanism, especially when drugs are not administered simultaneously, remains controversial [68].

NTBI is not only a more congenial diagnostic marker of iron overload and cellular toxicity than liver iron concentration or T2*MRI, but it is also a clinical parameter for monitoring chelation efficacy. LPI levels, in contrast, fluctuate based on the timing of chelator administration. The subcutaneous chelator DFO has a short half-life, limiting its impact on NTBI and LPI levels [69]. Therefore, it is recommended that oral chelators not be taken in the morning before blood sampling, while subcutaneous DFO should be administered overnight to maintain effectiveness [70].

The appearance of NTBI in plasma hastens iron deposition in other tissues, specifically in excitable tissues that contain Ca^2+^ channels, known to conduct Fe^2+^ ions into cells [71]. The abundance of functional voltage-gated Ca^2+^ channels in cardiac tissue makes the heart especially vulnerable to iron overload. Additionally, divalent metal transporters such as ZIP8 and ZIP14 also mediate Fe^2+^ uptake [72]. This iron accumulation promotes myocardial lipid peroxidation, impairs the activity of inner mitochondrial membrane respiratory enzymes, damages thiol-containing sarcolemmal enzymes such as 5′-nucleotidase and Na^+^/K^+^-ATPase, and disrupts cardiac contractility [73]. As such, cardiac cells are among the most susceptible to iron-induced toxicity, and cardiac complications from transfusion-related siderosis remain the most critical cause of mortality in thalassemia patients [74].

### 4.2. Potential Role of Antioxidants

Iron plays a central role in ROS generation, suggesting that iron chelators may also function as antioxidants. Iron chelation remains a key therapeutic strategy in IO disorders. The orally administered iron chelator DFP, known for its high membrane permeability, has been shown to remove free iron from β-thalassemia red blood cell membranes in a dose-dependent manner both in vivo and in vitro. DFP has also been reported to reduce iron-mediated membrane damage, including lipid peroxidation (as indicated by elevated malondialdehyde levels) and hemichrome formation [73]. However, DFP’s antioxidant effect alone is insufficient to fully prevent ROS-induced damage, as hemoglobin (Hb) levels improved in only a subset of patients.

A link between NTBI levels and lipid membrane oxidation has also been demonstrated through correlations between NTBI and malondialdehyde [75]. Efforts to enhance iron chelation with antioxidant supplementation have shown promise [76]. Some compounds exhibit dual properties as both iron chelators and antioxidants, including various polyphenols [77] and antioxidant-rich nutritional supplements such as curcumin and fermented papaya preparations [78,79]. Furthermore, the administration of iron-free (apo) transferrin has been shown to reduce iron overload symptoms in β-thalassemic mice [80], likely by binding NTBI. Vitamin E, a lipid-soluble antioxidant, has also been shown to restore oxidative balance and reduce ROS levels in the plasma [77].

Iron’s redox cycling depends on the presence of reducing agents that regenerate Fe^2+^ from Fe^3+^. In laboratory assays of LPI, this reducing function is provided by ascorbate, which facilitates the redox cycle at low concentrations. However, ascorbate exhibits both pro-oxidant and antioxidant behavior, depending on its relative concentration compared to redox-active metals. In vivo, ascorbate also scavenges ROS and regenerates other antioxidants [81]. LPI measurements are influenced by both iron content and antioxidant activity. Since these measurements are performed on properly stored serum or plasma samples, they reflect a balance between pro-oxidant iron and endogenous antioxidant defenses.

The total antioxidant capacity in human plasma is typically around 1 mM, but it is affected by factors such as diet and disease. Therefore, sera with similar NTBI concentrations may display different LPI levels depending on antioxidant content.

Thalassemia patients generally have lower plasma levels of antioxidants like ascorbate and vitamin E [11], along with increased levels of lipid peroxidation products such as malondialdehyde. Esposito et al. [11] demonstrated a correlation between NTBI and LPI levels, supporting the hypothesis that NTBI includes chemically labile iron detectable via physiological concentrations of ascorbate. This correlation may be obscured by the presence of antioxidants, suggesting that LPI is particularly useful as a biomarker in individuals with diminished antioxidant defenses, such as thalassemia patients or compromised neonates. However, caution is advised when administering high doses of ascorbate to such patients, as this may increase oxidative stress unless accompanied by iron chelation therapy.

## 5. Conclusions

Iron’s tendency to participate in Fenton chemistry is normally blocked by transferrin, which binds iron tightly and renders it non-labile [43]. In conditions such as hypotransferrinemia or systemic iron overload, this protective mechanism is overwhelmed, leading to the appearance of NTBI in plasma. NTBI is thought to be chemically labile, capable of redox activity and tissue uptake. Elevated markers of oxidative stress in iron-overloaded patients [2], the incorporation of labile iron into red blood cell membranes [73], and iron deposition in various tissues support this. However, experimental evidence for NTBI’s lability largely depends on its chelation behavior [76], which varies with the type of iron overload.

NTBI and other markers such as LPI and downstream indicators like LCI in specific tissues offer clinically useful information. These may aid in the early diagnosis of iron overload and the assessment of chelation efficacy, especially in maintaining plasma free of LPI. LPI has been evaluated as a surrogate marker for the effectiveness of chelation therapies, particularly regarding cardiac iron removal and function improvement [44]. Recent studies suggest that LPI closely reflects total body iron stores, such as liver iron content, in real time [45]. However, the clinical utility of NTBI and LPI as biomarkers depends on the development and validation of standardized assays [8].

NTBI and LPI are considered chemically reactive and contribute to oxidative stress participating in free-radical-generating reactions. However, LPI concentration in the bloodstream may be differentially affected by the mode and extent of iron overload, the presence of residual serum iron-binding activity, and the antioxidant capacity of individual sera [11]. In summary, both NTBI and LPI contribute to iron-mediated toxicity, but differ in terms of reactivity, availability, and pathogenic potential depending on the pathophysiological conditions that influence the degree of toxicity.

## Abbreviations

The following abbreviations are used in this manuscript.

NTBI	Non-transferrin-bound iron
LPI	Labile plasma iron
LCI	Labile cell iron
ROS	Reactive oxygen species
TDT	Transfusion-dependent thalassemia
NTDT	Non-transfusion-dependent thalassemia
MDS	Myelodysplastic syndrome
DFO	Deferoxamine
DFP	Deferiprone
DFX	Deferasirox
IO	Iron overload
